# Clinical Significance of Sarcopenia Defined by the Cross-Sectional Area of the Masseter Muscle in Cerebrovascular Events: A Retrospective Cohort Study

**DOI:** 10.3390/medicina61020268

**Published:** 2025-02-04

**Authors:** Fatih Seğmen, Semih Aydemir, Temel Kayan, Firdevs Tuğba Bozkurt Biçer, Cihangir Doğu, Esra Yakışık Aktekin, Deniz Erdem, Elif Uzun Ata

**Affiliations:** 1 Department of Intensive Care Unit, Ankara City Hospital, University of Health Sciences, 06800 Ankara, Turkey; drsegmen@gmail.com (F.S.); temel_kayan@hotmail.com (T.K.); drtugbaicu@gmail.com (F.T.B.B.); cihangirdogu@gmail.com (C.D.); pavulonmouse@hotmail.com (E.Y.A.); dh2erdem@gmail.com (D.E.); 2 Department of Anesthesiology and Reanimation, Yenimahalle Training and Research Hospital, University of Yıldırım Beyazit, 06370 Ankara, Turkey; 3 Department of Radiology, Ankara City Hospital, University of Health Sciences, 06800 Ankara, Turkey; uzunata@gmail.com

**Keywords:** cerebrovascular events, sarcopenia, masseter muscle, cross-sectional area, stroke, mortality, nutritional status, intensive care

## Abstract

*Background and Objectives*: This study aimed to investigate the clinical significance of sarcopenia, defined by the cross-sectional area of the masseter muscle (CSA-M), as an early marker for sarcopenia diagnosis and its association with mortality in patients with cerebrovascular events (CVE). *Materials and Methods:* In this retrospective cohort study, 120 patients aged 65 years or older with CVE admitted to Bilkent City Hospital between September 2020 and September 2023 were included. Patients with malignancy, prior CVE, or incomplete data were excluded. Parameters such as CSA-M measured via brain CT, Acute Physiology and Chronic Health Evaluation II (APACHE II) and Sequential Organ Failure Assessment (SOFA) scores, Nutritional Risk Score (NRS), duration of ICU and hospital stays, and 28-day mortality were evaluated. The CSA-M thresholds for sarcopenia were defined as <400 mm^2^ for men and <300 mm^2^ for women. *Results:* Sarcopenia prevalence was significantly associated with prolonged ICU (27.0 ± 33.1 days vs. 16.5 ± 22.4 days, *p* = 0.042) and hospital stays (34.8 ± 38.4 days vs. 21.3 ± 22.3 days, *p* = 0.017). Right and left CSA-M values were significantly lower in sarcopenic patients (*p* < 0.001). ROC analysis revealed CSA-M cut-off values of <300 mm^2^ (AUC = 0.82) for men and <295 mm^2^ (AUC = 0.83) for women as strong predictors of sarcopenia. Multivariate regression analysis showed a significant association between CSA-M and 28-day mortality (*p* < 0.05). Sarcopenia also correlated with lower albumin levels, a higher prevalence of ischemic stroke, and increased mechanical ventilation needs. *Conclusions:* CSA-M measured via brain CT is a reliable marker for sarcopenia and a predictor of clinical outcomes in CVE patients. Early identification and management of sarcopenia could improve patient prognosis. Further research is warranted to explore its potential in broader clinical contexts.

## 1. Introduction

Stroke is a significant cerebrovascular event resulting from a sudden interruption or reduction in cerebral blood flow, associated with high morbidity and mortality. With the rapid aging of the global population, the incidence of stroke has risen, with approximately 13 million new cases reported annually. Among these, ischemic strokes are the most prevalent, followed by hemorrhagic strokes [1].

Sarcopenia, a syndrome characterized by progressive loss of skeletal muscle mass and strength, commonly develops with aging and contributes to falls, fractures, reduced quality of life, and increased morbidity and mortality [2,3]. The prevalence of sarcopenia can reach up to 56% among hospitalized patients [4]. Studies in intensive care unit (ICU) settings have linked sarcopenia to poor outcomes, including increased mortality, highlighting the importance of assessing muscle mass and function in critically ill patients [5,6].

Stroke-related sarcopenia refers to systemic muscle mass loss and functional decline following a stroke [7,8]. The acute phase of stroke is frequently accompanied by motor paralysis or impaired consciousness, resulting in a rapid reduction of motor units and a significant loss of muscle mass, which can begin as early as four hours post-stroke [4]. Although the exact mechanisms underlying stroke-related sarcopenia remain unclear, factors such as malnutrition, sympathetic overactivation, spasticity, denervation, immobilization, and dysfunctional atrophy are thought to contribute [7,9,10]. Low muscle mass at stroke onset and the presence of stroke-related sarcopenia have been associated with increased mortality [7,11]. Despite its clinical significance, data on the prevalence of stroke-related sarcopenia are limited, with reported rates ranging between 16.5% and 56.4%. This prevalence is expected to rise over the coming decades [4,7].

Sarcopenia diagnosis requires the evaluation of muscle mass and function. Imaging modalities such as MRI, CT, dual-energy X-ray absorptiometry (DXA), and ultrasonography (USG) are commonly employed to assess muscle quality and lean body mass. MRI is considered the gold standard for evaluating total muscle mass and quality, while CT is the preferred method for assessing lean muscle mass, despite its associated cost, accessibility challenges, and radiation exposure [3]. The 2019 update by the European Working Group on Sarcopenia in Older People (EWGSOP2) emphasized muscle strength as the most reliable indicator of muscle function, noting that it is more sensitive than muscle mass in predicting prognosis [3]. However, assessing muscle strength and performance in stroke patients is challenging due to factors such as altered consciousness, disorientation, and sedation [4,7].

Among the various muscles assessed for sarcopenia, the masseter muscle offers unique advantages due to its accessibility and reproducibility in measurement via routine cranial imaging, such as brain CT scans. Unlike limb muscles, which may require specialized imaging modalities like MRI or DXA, the masseter muscle can be evaluated in a non-invasive and cost-effective manner in acute care settings. Additionally, the masseter muscle plays a critical role in mastication and nutritional intake, making it a functional, as well as structural, indicator of overall muscle health. These characteristics make the masseter muscle a promising and practical marker for sarcopenia, particularly in cerebrovascular event patients [4,7].

The distinction between primary and secondary sarcopenia is critical in understanding the etiology and management of this condition. Primary sarcopenia is attributed to aging and the physiological decline in muscle mass and function, whereas secondary sarcopenia arises from underlying conditions such as stroke, chronic inflammation, malnutrition, or prolonged immobilization. Stroke-induced secondary sarcopenia is often characterized by asymmetrical muscle loss and functional impairments due to neurological deficits. While stroke-related sarcopenia contributes to systemic muscle mass loss and functional decline, distinguishing between primary and secondary sarcopenia in cerebrovascular event (CVE) patients remains challenging and underexplored. Studies specifically addressing primary sarcopenia in CVE patients are limited, emphasizing the need for further research to tailor interventions effectively [7,8].

Emerging evidence suggests a relationship between the masseter muscle, nutritional status, and whole-body skeletal muscle mass [12,13]. Notably, the cross-sectional area of the masseter muscle, measurable via head CT, has been proposed as a marker for sarcopenia. A decreased masseter muscle area has been associated with higher two-year mortality following trauma [14]. Recent studies further indicate that sarcopenia diagnosed through masseter muscle assessment correlates with early mortality in patients with stable traumatic brain injury [15,16]. However, this association remains poorly defined in patients with CVE.

Despite significant advancements in understanding sarcopenia and its implications in critically ill patients, there remains a lack of data exploring the potential of localized muscle assessments, such as the cross-sectional area of the masseter muscle (CSA-M), as a reliable early marker for sarcopenia. Most studies focus on systemic assessments, which are often challenging to apply in stroke patients due to their clinical status [7,8]. This gap in the literature, combined with the increasing need for efficient and accessible diagnostic tools, motivated this study. Additionally, understanding the association between CSA-M and outcomes in CVE patients could provide critical insights for early intervention strategies, potentially improving prognosis and resource allocation in intensive care settings.

In this study, we aim to investigate whether sarcopenia, as assessed by the CSA-M on initial brain CT, serves as an early marker for predicting primary sarcopenia and mortality in CVE patients.

## 2. Materials and Methods

### 2.1. Study Design

This study was designed as a single-center retrospective cohort study. The study included patients aged 65 and over who were admitted to Bilkent City Hospital between September 2020 and September 2023 with a diagnosis of CVE and who were admitted to the emergency department, underwent brain CT, and were admitted to the intensive care unit (ICU). Patients with a history of malignancy, patients whose follow-up data and imaging were not available, and patients who had previously had CVE were not included in the study.

Within the scope of the study, parameters such as age, gender, APACHE II score, SOFA score, Nutritional Risk Score (NRS), CVE etiology, treatment method, mechanical ventilation requirement, masseter muscle cross-sectional area (with distinction between men and women), length of stay in intensive care and hospital, and 28-day mortality rate of the patients were evaluated.

To ensure the quality and reliability of the data collected from medical records, a multi-step validation process was implemented. Two independent researchers reviewed the medical records to extract key variables, including CSA-M measurements, APACHE II, and SOFA scores, to minimize data entry errors. Any discrepancies were resolved through consensus or consultation with a third senior researcher. Furthermore, predefined inclusion and exclusion criteria were strictly applied to avoid confounding factors. The primary outcomes were validated by cross-referencing data with electronic health records and radiological imaging reports. These measures ensured the accuracy and consistency of the retrospective data utilized in this study.

### 2.2. APACHE II (Acute Physiology and Chronic Health Evaluation II) Score

The APACHE II score is a widely used severity-of-disease classification system in intensive care units. It includes 12 physiological variables (e.g., body temperature, heart rate, respiratory rate, and arterial blood gas values), as well as patient age and chronic health status. Each parameter is scored, and the total score predicts the mortality risk, with higher scores indicating a greater severity of illness [17,18].

### 2.3. SOFA (Sequential Organ Failure Assessment) Score

The SOFA score evaluates the extent of a patient’s organ dysfunction or failure across six organ systems: respiratory, cardiovascular, hepatic, coagulation, renal, and central nervous system. Each organ system is assigned a score from 0 (normal function) to 4 (severe dysfunction), with the total score reflecting overall organ dysfunction severity [19].

### 2.4. Nutritional Risk Score (NRS)

The Nutritional Risk Score is designed to assess the nutritional status of hospitalized patients. It combines the severity of malnutrition (based on body mass index, weight loss, or dietary intake) with the severity of disease. Scores above 3 indicate a high risk of malnutrition, warranting immediate nutritional intervention [20,21].

### 2.5. Cross-Sectional Area of the Masseter Muscle (CSA-M)

The masseter muscle cross-sectional area was calculated from the patients’ brain CT images. There is no generally accepted specific cut-off value for sarcopenia defined by the masseter muscle cross-sectional area, as this measurement may vary depending on age, gender, body mass, and other individual differences. However, several studies have attempted to predict sarcopenia using specific cut-off values for the masseter muscle cross-sectional area measurement. For example, studies on brain CT have generally considered the masseter muscle cross-sectional area to be around 400 mm^2^ in men and around 300 mm^2^ in women when normal [22,23,24]. Individuals with cross-sectional area values below these limits are considered to have decreased muscle mass (sarcopenia) [22,23,24]. However, the diagnosis of sarcopenia is not limited to cross-sectional area measurement alone but is evaluated together with other parameters such as muscle function and strength.

### 2.6. Statistical Analysis

All statistical analyses were performed using SPSS version 27.0 (IBM Corp., Armonk, NY, USA). The sample size was calculated to detect a statistically significant association between CSA-M and 28-day mortality in CVE patients, with an expected effect size of 0.35, a statistical power of 80%, and a significance level of 0.05. Based on these parameters, a minimum sample size of 88 participants was required. To account for potential data loss or exclusions, the final sample size was increased to 120 patients. Continuous variables were presented as the mean ± standard deviation (SD) and compared using the independent samples *t*-test or Mann–Whitney *U* test based on data distribution assessed by the Kolmogorov–Smirnov and Shapiro–Wilk tests. Categorical variables were expressed as frequencies (%) and compared using the chi-square test or Fisher’s exact test. Correlations between the CSA-M and clinical parameters were evaluated using Pearson’s or Spearman’s correlation coefficients. ROC curve analysis determined the CSA-M cut-off values for sarcopenia, reporting sensitivity, specificity, and area under the curve (AUC). Predictors of sarcopenia and 28-day mortality were identified through univariate and multivariable logistic regression analyses, with significant variables (*p* < 0.1) from the univariate analysis included in the multivariable model. The results were considered statistically significant at *p* < 0.05.

## 3. Results

The socio-demographic and clinical parameters of patients are shown in Table 1. A total of 120 patients were included in the study. The median age was 81 (74–86) years. Hypertension (83.3%) was the most common comorbidity, followed by coronary artery disease (35.8%) and hyperlipidemia (37.5%). Other notable comorbidities included dementia (38.3%), atrial fibrillation (16.7%), and chronic renal failure (3.3%). Ischemic stroke was observed in 83.3% of the patients, and hemorrhagic stroke was observed in 15.8%. The mean SOFA score was 7.2 ± 3.4, and the APACHE II score was 24.3 ± 7.2. The median albumin value was 30.5 (27–35) g/dL, and the total protein value was 57.7 ± 8.3 mg/dL. The mean lymphocyte count was 0.90 ± 0.5 (10^9^/L). The mean CRP was 91.7 ± 83.6 mg/dL. The mean cross-sectional area of the right masseter muscle was 322.1 ± 84.2 mm^2^, and the left masseter muscle was 318.1 ± 86.5 mm^2^. The mean length of stay in the intensive care unit was 23.5 (10–60) days, the length of hospital stay was 30.2 ± 34.3 days. 32.5% of the patients died within 28 days, and the total mortality rate was 54.2% (Table 1).

Comparison of the parameters according to sarcopenia status is shown in Table 2. While the right mean CSA-M was 399.9 ± 75.7 mm^2^ and the left mean was 399.6 ± 72.2 mm^2^ in patients without sarcopenia, the right mean was 281.8 ± 55.2 mm^2^ and the left mean was 275.8 ± 58.8 mm^2^ in patients with sarcopenia, and these differences were found to be statistically significant (*p* < 0.001 and *p* < 0.001). The mean age of patients with sarcopenia (81.7 ± 8.3 years) was found to be significantly higher than that without sarcopenia (77.8 ± 9.4 years) (*p* = 0.023). The albumin level in patients without sarcopenia (33.5 (22.7–44.3 g/dL)) was found to be significantly higher than in patients with sarcopenia (31.1 (19.3–42.9 g/dL)) (*p* = 0.033). The mean duration of intensive care unit stay in patients without sarcopenia was measured as 16.5 ± 22.4 days, and in patients with sarcopenia, it was measured as 27.0 ± 33.1 days, and this difference was found to be statistically significant (*p* = 0.042). The mean duration of hospital stay in patients without sarcopenia was found to be 21.3 ± 22.3 days, and in patients with sarcopenia, it was found to be 34.8 ± 38.4 days, and this difference was also significant (*p* = 0.017). No significant difference was found between the two groups in terms of SOFA (*p* = 0.593) and APACHE II (*p* = 0.973) scores. There was no significant difference between the two groups in terms of lymphocyte count, CRP, total protein, and PNI (*p* > 0.05). There was no significant relationship between sarcopenia status and 28-day mortality (*p* = 0.069). The rate of ischemic stroke was found to be higher in patients with sarcopenia (*p* = 0.041). The prevalence of dementia was significantly higher in patients with sarcopenia (*p* = 0.030). There was no significant difference between the groups in terms of mechanical ventilation use (*p* = 0.060) (Table 2).

The mean CSA-M measurements for the left and right sides are summarized in Table 3. While the mean CSA-M of the right side was measured as 458.5 ± 39.5 mm^2^ and the left side was 446.3 ± 46.7 mm^2^ in men without sarcopenia, the right-side mean was 302.5 ± 56.8 mm^2^ and left-side mean was 301.3 ± 57.7 mm^2^ in men with sarcopenia (*p* < 0.001). The mean age of men with sarcopenia was 80.4 ± 8.2 years and 74.2 ± 12.6 years in those without, and the difference was significant (*p* = 0.039). The duration of intensive care unit stay was calculated as 28.5 ± 32.5 days in those with sarcopenia and 12.2 ± 17.5 days in those without (*p* = 0.024). The duration of hospital stay was measured as 35.2 ± 35.4 days in those with sarcopenia and 14.9 ± 17.4 days in those without sarcopenia, and the difference was significant (*p* = 0.007). There was no statistically significant difference between the groups in terms of total protein, albumin, CRP, lymphocyte count, and PNI (*p* > 0.05). No significant difference was found between sarcopenia and 28-day mortality (*p* = 0.843). No significant association was found between sarcopenia and total mortality (*p* = 0.272). Ischemic stroke (85.4%) was more common in those with sarcopenia, while the rate of hemorrhagic stroke (14.6%) was lower (*p* = 0.108). No significant difference was found between the two groups in terms of the use of mechanical ventilation (*p* = 0.183) (Table 3).

Comparison of the parameters according to sarcopenia status (CSA-M < 300) in women is shown in Table 4. While the mean CSA-M of the right side was 375.7 ± 74.2 mm^2^ and the left side was 380.2 ± 72.6 mm^2^ in women without sarcopenia, the mean CSA-M of the right side was 249.7 ± 33.3 mm^2^ and the left side was 236.3 ± 33.4 mm^2^ in women with sarcopenia, and the difference was found to be statistically significant (*p* < 0.001 and *p* < 0.001). The mean age of women with sarcopenia was 83.5 ± 8.2 years and 79.3 ± 7.4 years in women without sarcopenia (*p* = 0.047). No significant difference was found between women with and without sarcopenia in terms of SOFA score, APACHE II score, and NRS (*p* > 0.05). Duration of Intensive Care Unit was found to be 24.7 ± 34.5 days in those with sarcopenia and 18.2 ± 24.3 days in those without (*p* = 0.407). Duration of Hospital Stay was measured as 34.1 ± 43.2 days in those with sarcopenia and 24.0 ± 23.9 days in those without, and the difference was not found to be significant (*p* = 0.261). No significant difference was found between women with and without sarcopenia in terms of total protein, albumin, CRP, lymphocyte count, and PNI (*p* > 0.05). No significant difference was found between women with and without sarcopenia in terms of 28-day mortality (*p* = 0.296). No significant difference was found between the two groups in terms of total mortality (*p* = 0.308). The rate of ischemic stroke (71%) was found to be higher in women with sarcopenia than in those without (*p* = 0.049). The use of mechanical ventilation was found to be higher in women with sarcopenia (*p* = 0.035) (Table 4).

A significant positive correlation was found between CSA-M and total protein level (r = 0.380, *p* = 0.010), albumin level (r = 0.520, *p* = 0.001), and lymphocyte count (r = 0.420, *p* = 0.005). There was also a significant positive correlation between CSA-M and PNI (r = 0.550, *p* = 0.001). Additionally, female sex showed a positive correlation with CSA-M (r = 0.250, *p* = 0.040). CSA-M was negatively correlated with age (r = −0.450, *p* = 0.001), SOFA score (r = −0.550, *p* = 0.001), APACHE II score (r = −0.600, *p* = 0.001), NRS (r = −0.500, *p* = 0.002), and ischemic CVE (r = −0.400, *p* = 0.015). The use of mechanical ventilation was also negatively correlated with CSA-M (r = −0.520, *p* = 0.001). Furthermore, there was a significant negative correlation between CSA-M and the length of stay in the ICU (r = −0.480, *p* = 0.002), as well as the length of hospital stay (r = −0.440, *p* = 0.004). CSA-M was negatively correlated with the CRP levels (r = −0.380, *p* = 0.004) and 28-day mortality (r = −0.620, *p* = 0.001) (Table 5).

The effect of the parameters in predicting sarcopenia is shown in Table 6. The results show that the CSA-M is a strong determinant in predicting sarcopenia (For right CSA-M, *p* = 0.018 in the univariate analysis and *p* = 0.044 in the multivariate analysis; for left CSA-M, *p* = 0.007 in the univariate analysis and *p* = 0.035 in the multivariate analysis). A significant relationship was found between age and sarcopenia (*p* = 0.045), and this relationship continued in the multivariate analysis (*p* = 0.050). No significant relationship was found between male sex and sarcopenia in the univariate analysis (*p* = 0.052), and no significance was observed in the multivariate analysis (*p* = 0.070). Ischemic CVE was found to be significant in both analyses (*p* = 0.024 and *p* = 0.046), suggesting that this event may be associated with sarcopenia. The NRS score, which evaluates nutritional status, was found to be significant in the univariate analysis (*p* = 0.048) but remained at the limit of significance in the multivariate analysis (*p* = 0.054). The total protein and albumin levels did not show a significant relationship in both analyses (*p* > 0.05). The PNI did not show a significant relationship in both the univariate and multivariate analyses (*p* > 0.05) (Table 6).

**Figure 1:**
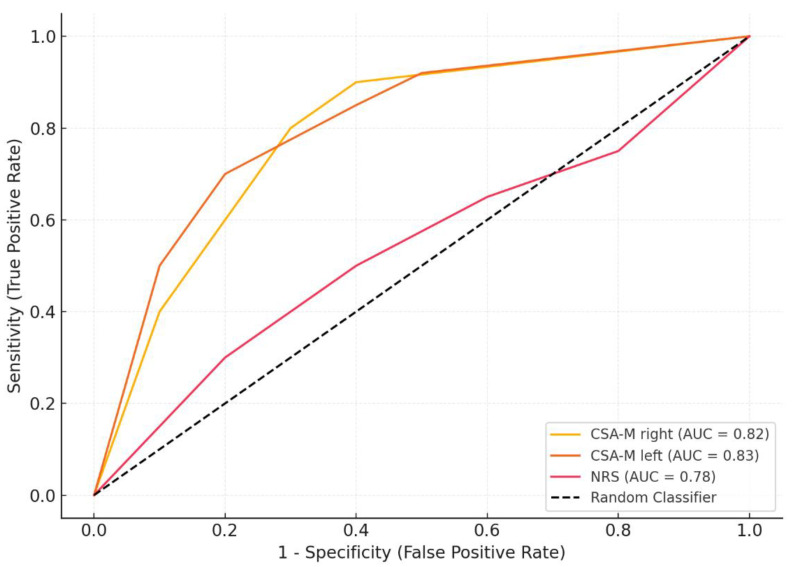
ROC analysis results in patients with sarcopenia.

The ROC analysis results in patients with sarcopenia are shown in Table 7. The cut-off point for CSA-M right was determined as <300 mm^2^, the sensitivity was 85%, the specificity was 78%, and the AUC was 0.82 (95% CI: 0.76–0.88) (*p* = 0.001). Similarly, the cut-off point for CSA-M left was determined as <295 mm^2^, the sensitivity was 87%, the specificity was 80%, and the AUC was 0.83 (95% CI: 0.77–0.89), making this parameter a strong indicator in predicting sarcopenia (*p* = 0.001). The cut-off point for NRS was determined as >5, the sensitivity was 75%, the specificity was 70%, and the AUC was 0.78 (95% CI: 0.70–0.85) (*p* = 0.004). These findings indicate that NRS may have an important role in predicting sarcopenia, although not as strong as CSA-M (Table 7, Figure 1).

The results of the univariate and multivariable regression analyses with 28-day mortality in sarcopenia patients are shown in Table 8. The right and left CSA-M were found to be significant in both univariate and multivariate analyses: for the right CSA-M, it was recorded as *p* = 0.018 in the univariate regression and *p* = 0.044 in the multivariate regression; for the left CSA-M, it was recorded as *p* = 0.007 in the univariate analysis and *p* = 0.035 in the multivariate analysis. While no significant association was found between age and mortality, ischemic CVE showed a significant association with *p* = 0.024 in the univariate analysis and *p* = 0.046 in the multivariate analysis. The use of mechanical ventilation was significantly associated with mortality in both analyses (*p* = 0.011 and *p* = 0.042), demonstrating the prognostic importance of mechanical ventilation. The SOFA score was found to be significant with *p* = 0.039 in the univariate analysis and *p* = 0.048 in the multivariate analysis, but no significant association was observed with the APACHE II score (*p* > 0.05). Parameters such as albumin and PNI were not associated with mortality. A significant association was found between the length of stay in the intensive care unit and 28-day mortality in both the univariate and multivariate analyses. In the univariate analysis, *p* = 0.032, and the effect size was calculated as 1.504 (95% CI: 1.307–1.702). In the multivariate analysis, *p* = 0.045, and effect size were determined as 1.399 (95% CI: 1.201–1.605). These results show that increasing the length of stay in intensive care may significantly increase the risk of 28-day mortality. For the duration of hospitalization, *p* = 0.046, and the effect size was found as 1.402 (95% CI: 1.203–1.604) in the univariate analysis. However, it was at the limit of significance with *p* = 0.055 in the multivariate analysis, and the effect size was calculated as 1.304 (95% CI: 1.102–1.506). Although these results suggest that the duration of hospitalization may be associated with mortality, the multivariate analysis results indicate that this association is weaker. These results reveal that, especially CSA-M and the SOFA score, may be important prognostic indicators in predicting mortality in sarcopenia patients (Table 8).

## 4. Discussion

This study examined the clinical significance of sarcopenia, as defined by the CSA-M, in patients with cerebrovascular events. The primary findings demonstrated a significant association between sarcopenia and extended lengths of hospital and ICU stays, as well as correlations with biochemical markers indicative of poor prognosis. Additionally, CSA-M was found to be an important marker in predicting mortality.

In our study, the mean age of patients in the sarcopenia group (81.7 ± 8.3 years) was found to be significantly higher. This finding is consistent with previous studies showing that aging contributes to the decrease in muscle mass [25,26,27]. Zhang et al. reported that aging is one of the main factors of the decrease in muscle mass and that the prevalence of sarcopenia increases in older individuals [26]. Our study confirms this relationship but emphasizes that sarcopenia increases the risk of complications with advanced age.

The significant correlations between CSA-M and key nutritional indicators, such as albumin levels and the PNI, emphasize the importance of nutritional interventions in managing sarcopenic patients [28,29]. Strategies aimed at optimizing the nutritional status and reducing systemic inflammation could potentially mitigate the adverse outcomes associated with sarcopenia in cerebrovascular patients. Furthermore, given the high prevalence of dementia in sarcopenic patients observed in our study, as corroborated by studies [30,31,32,33], a multidimensional management approach addressing both cognitive and physical health is warranted. In our study, the albumin levels were found to be significantly lower in the sarcopenia group (31.1 ± 5.9 g/dL). This finding is consistent with the study by Charlton et al., which showed that inflammation and malnutrition are associated with low albumin levels, and this may lead to poor clinical outcomes [34]. Albumin is commonly used as an indicator of nutritional status and general health; however, its interpretation must consider the influence of inflammation, as elevated CRP levels during the inflammatory stage can lower the albumin levels, irrespective of nutritional status [35]. Our study demonstrates that albumin levels are valuable not only in the diagnosis of sarcopenia but also in the evaluation of clinical outcomes.

The association between sarcopenia and adverse clinical outcomes in cerebrovascular diseases has been documented in several studies [36,37,38]. Kim et al. reported that sarcopenia, as determined by skeletal muscle mass measurements, was a strong predictor of prolonged hospital stays and higher mortality rates in stroke patients [39]. Similarly, in our cohort, patients with sarcopenia exhibited significantly longer ICU and hospital stays compared to those without sarcopenia. The negative correlation observed between CSA-M and length of stay further underscores the role of muscle mass as a determinant of recovery duration. In our study, patients in the sarcopenia group were found to have longer hospital stays (34.8 ± 38.4 days) and in the intensive care unit (27.0 ± 33.1 days). Rolland et al. reported that sarcopenia patients had 30–50% longer hospital stays than healthy individuals [37]. Our results suggest that there is a link between sarcopenia and an increased risk of complications, supporting that this association results in greater use of healthcare resources. These findings suggest that addressing sarcopenia early in the course of cerebrovascular disease management could shorten recovery periods and improve outcomes.

In our study, CSA-M was shown to be a strong marker in the diagnosis of sarcopenia. CSA-M values of the right and left-side were found to be significantly lower in the sarcopenia group (*p* < 0.001). Gonzalez et al. emphasized that CSA-M is an effective method for assessing muscle mass [40]. Wallace et al. stated that CSA-M is an effective method for assessing sarcopenia in elderly patients [14]. In our study, CSA-M was found to have high sensitivity and specificity, with cut-off values of <300 mm^2^ for the right side and <295 mm^2^ for the left side (AUC: 0.82 and 0.83). These findings suggest that CSA-M can be used not only as a diagnostic but also as a prognostic marker. Our study expands the limited data in the literature and highlights the importance of CSA-M in clinical practice.

In terms of mortality, while no significant difference in 28-day mortality was observed between sarcopenic and non-sarcopenic groups, this contrasts with the findings of Cruz-Jentoft et al., who reported sarcopenia as an independent predictor of short-term mortality in stroke patients [3]. This discrepancy may stem from differences in the criteria used to define sarcopenia. Our study’s use of CSA-M as a localized measurement focuses on a specific anatomical site, which may not fully capture the systemic impact of sarcopenia. In our study, low CSA-M showed a significant negative association with mortality (*r* = −0.620, *p* < 0.001). This finding is consistent with the study by Gonzalez et al. indicating that low muscle mass is associated with an increased mortality risk [40]. The usability of the masseter muscle in mortality prediction has been studied little in the literature, and in this context, our study makes an important contribution to the literature.

In our study, the ischemic stroke rate (85.4%) was found to be significantly higher in the sarcopenia group. The observed higher prevalence of ischemic stroke in sarcopenic patients is consistent with findings by Yoshimura et al., who suggested that sarcopenia might increase the vulnerability to ischemic cerebrovascular events through systemic inflammation and impaired vascular integrity [25]. Our study shows a significant negative correlation between CSA-M and inflammatory markers such as CRP, as well as a positive correlation between CSA-M and nutritional indices, including albumin and PNI. However, it is important to note that elevated CRP levels, indicative of a higher inflammatory state, may influence albumin levels independently of the nutritional status. Therefore, caution must be exercised in interpreting albumin as a sole indicator of nutrition during inflammatory conditions. These results emphasize the intertwined relationship between sarcopenia, inflammation, and malnutrition. Liao et al. showed that sarcopenia may increase the risk of ischemic stroke through increased inflammatory processes and vascular dysfunction [41]. Our study reveals that this relationship is clearly seen in the sarcopenia group and that the potential mechanisms between sarcopenia and stroke should be further investigated.

Inflammation plays a pivotal role in muscle loss, particularly in sarcopenia associated with CVE. While our study included CRP as a general marker of inflammation, and integrating more specific biomarkers, such as interleukin-6 (IL-6), tumor necrosis factor-alpha (TNF-α), and myostatin, could provide deeper insights into the inflammatory mechanisms contributing to muscle wasting. These biomarkers have been shown to be closely linked to muscle catabolism and sarcopenia in previous studies, and their inclusion in future research could enhance our understanding of the relationship between inflammation and sarcopenia.

In our study, it was observed that the rate of mechanical ventilation use was higher in the sarcopenia group (30%, *p* = 0.060). In the literature, sarcopenia has been reported to reduce the strength of the respiratory muscles and increase the need for ventilation [42]. Our study confirms this connection and emphasizes that the need for ventilation should be taken into account in the prognosis of sarcopenia.

Our study provides important contributions to the literature by emphasizing the importance of CSA-M in the diagnosis of sarcopenia and the prediction of clinical outcomes. In particular, considering the limited literature on the relationship between CSA-M and mortality and other clinical outcomes, these findings are of original value. Another important contribution of our study is that the cross-sectional area of the masseter muscle was evaluated by directly measuring it with brain CT. This method offers a non-invasive and rapid assessment. Similarly, the relationship between CSA-M and the need for mechanical ventilation and the duration of intensive care increases the potential of this method for use in clinical decision-making processes.

While we acknowledge that gender-based subgroup analysis may reduce statistical power, it was included due to known physiological differences in muscle mass and distribution between men and women, which may influence CSA-M values. Gender-specific thresholds for sarcopenia diagnosis are widely recognized in the literature, and separating the data by gender allowed us to evaluate the applicability of these thresholds in our cohort. However, we agree that further studies with larger, multi-center cohorts are necessary to validate these findings and strengthen the generalizability of the results.

### 4.1. Clinical Implications of Findings

The findings of this study have significant implications for clinical practice. First, the CSA-M, as a reliable and easily measurable marker using routine brain CT scans, offers a non-invasive and rapid method for identifying sarcopenia in CVE patients. Early identification of sarcopenia through CSA-M measurements allows clinicians to implement timely nutritional and physical rehabilitation interventions, potentially reducing hospital and ICU stays. Furthermore, given the association of sarcopenia with adverse outcomes, routine CSA-M assessment in high-risk patients could improve prognostic stratification and guide resource allocation, especially in critical care settings. Healthcare providers could also use CSA-M measurements to monitor the efficacy of the interventions aimed at improving muscle mass and overall patient outcomes. Future guidelines may consider incorporating CSA-M as a standard assessment tool for sarcopenia in CVE patients.

An important aspect to consider is the economic impact of increased hospital and ICU stays in sarcopenic patients. Sarcopenia-associated functional decline and poorer clinical outcomes may lead to prolonged hospitalization, higher ICU costs, and increased utilization of healthcare resources. These economic burdens underline the importance of early detection and intervention strategies, such as nutritional support and physical rehabilitation, which could potentially reduce healthcare costs and improve patient outcomes. Further research exploring the cost-effectiveness of such interventions is recommended.

### 4.2. Limitations of the Study

This study has several limitations. Its single-center and retrospective design may introduce selection bias and limit the generalizability of the findings. The retrospective nature of the study prevents the establishment of causal relationships between variables, allowing only for the identification of correlations and hypothesis generation. Furthermore, the use of CSA-M as the sole indicator of sarcopenia may not fully capture the multifaceted nature of this condition, which encompasses both muscle mass and function. Functional parameters such as handgrip strength or gait speed were not assessed, further limiting the scope of the findings.

Despite these limitations, the study has notable strengths. The use of CSA-M as a straightforward and reproducible parameter provides a practical approach to defining sarcopenia, particularly in settings where whole-body muscle mass assessment is not feasible. Additionally, the retrospective design enabled the analysis of a substantial patient cohort, offering robust statistical insights. Future prospective studies incorporating functional assessments are warranted to validate and expand upon these findings and to better understand the predictive value and causal pathways associated with CSA-M.

## 5. Conclusions

In conclusion, this study underscores the clinical relevance of sarcopenia, as defined by CSA-M, in predicting adverse outcomes in cerebrovascular events. The findings highlight the importance of early identification and targeted management of sarcopenia to improve patient outcomes. Further research is needed to elucidate the underlying mechanisms linking sarcopenia to inflammation and cerebrovascular outcomes, as well as to evaluate the utility of CSA-M in broader clinical contexts.

## Figures and Tables

**Table 1:** Socio-demographic and clinical parameters of the patients.

Parameters	Patients (N = 120) n (%), Mean ± SD
Gender	
Male	60 (50.0%)
Female	60 (50.0%)
Age (Year)	81 (74–86)
Comorbid Diseases	
Osteoporosis	1 (0.8%)
Hypertension	100 (83.3%)
Coronary artery disease	43 (35.8%)
Hyperlipidemia	45 (37.5%)
Dementia	46 (38.3%)
Heart failure	14 (11.7%)
Atrial fibrillation	20 (16.7%)
Pulmonary arterial hypertension	10 (8.3%)
Parkinson’s	2 (1.7%)
Chronic renal failure	4 (3.3%)
Scleroderma	1 (0.8%)
Benign prostatic hyperplasia	1 (0.8%)
Rheumatoid arthritis	1 (0.8%)
Cerebrovascular event (CVE)	
Hemorrhagic	19 (15.8%)
Ischemic	101 (84.2%)
SOFA	7.2 ± 3.4
APACHE II	24.3 ± 7.2
NRS	5.9 ± 1.4
CSA-M (mm^2^)	
Right	322.1 ± 84.2
Left	318.1 ± 86.5
Total protein (mg/dL)	57.7 ± 8.3
Albumin (g/dL)	30.5 (27–35)
CRP (mg/dL)	91.7 ± 83.6
Lymphocyte (10^9^/L)	0.90 ± 0.5
PNI (Lymphocyte × Albumin)	29.0 ± 17.0
Use of Mechanical Ventilation	36.0 (30.0%)
Duration of stay in intensive care (Day)	23.5 (10–60)
Duration of stay in hospital (Day)	30.2 ± 34.3
28-day mortality	39 (32.5%)
Mortality	65 (54.2%)

CSA-M: cross-sectional area of the masseter muscle, SOFA: Sequential Organ Failure Assessment, APACHE II: Acute Physiology and Chronic Health Evaluation II, NRS: Nutritional Risk Score, and PNI: Prognostic Nutritional Index.

**Table 2:** Comparison of the parameters according to sarcopenia status.

Parameters	Sarcopenia No (n = 41)	Sarcopenia Yes (n = 79)	*p* Value
CSA-M (mm^2^)			
Right-side	399.9 ± 75.7	281.8 ± 55.2	**<0.001**
Left-side	399.6 ± 72.2	275.8 ± 58.8	**<0.001**
Age (Year)	77.8 ± 9.4	81.7 ± 8.3	**0.023**
SOFA	7.1 ± 3.7	7.3 ± 3.3	0.593
APACHE II	24.0 ± 7.3	24.5 ± 7.2	0.973
NRS	5.6 ± 1.7	6.1 ± 1.2	0.065
Duration of stay in intensive care (Day)	16.5 ± 22.4	27.0 ± 33.1	**0.042**
Duration of stay in hospital (Day)	21.3 ± 22.3	34.8 ± 38.4	**0.017**
Total protein (mg/dL)	59.4 ± 7.6	56.8 ± 8.6	0.109
Albumin (g/dL)	33.5 (22.7–44.3)	31.1 (19.3–42.9)	**0.033**
CRP (mg/dL)	85.4 ± 98.9	94.9 ± 75.1	0.053
Lymphocyte (10^9^/L)	0.87 ± 0.4	0.92 ± 0.6	0.905
PNI (Lymphocyte × Albumin)	29.1 ± 16.5	28.9 ± 17.3	0.877
Dementia			
No	31	43	**0.030**
Yes	10	36	
28-day mortality			
No	26	55	0.629
Yes	15	24	
Mortality			
No	24	31	0.069
Yes	17	48	
Cerebrovascular event (CVE)			
Hemorrhagic	10	9	**0.041**
Ischemic	31	70	
Use of Mechanical Ventilation			
No	31	53	0.060
Yes	10	26	

CSA-M: cross-sectional area of the masseter muscle, SOFA: Sequential Organ Failure Assessment; APACHE II: Acute Physiology and Chronic Health Evaluation II, NRS: Nutritional Risk Score, and PNI: Prognostic Nutritional Index. Statistically significant *p* values are in bold.

**Table 3:** Comparison of the parameters according to sarcopenia status (CSA-M < 400) in men.

Parameters	Sarcopenia No (n = 12)	Sarcopenia Yes (n = 48)	*p* Value
CSA-M (mm^2^)			
Right-side	458.5 ± 39.5	302.5 ± 56.8	**<0.001**
Left-side	446.3 ± 46.7	301.3 ± 57.7	**<0.001**
Age (Year)	74.2 ± 12.6	80.4 ± 8.2	**0.039**
SOFA	9.6 ± 4.7	7.8 ± 3.1	0.221
APACHE II	27.7 ± 8.8	24.6 ± 7.8	0.286
NRS	6.0 ± 1.7	6.0 ± 1.2	0.876
Duration of stay in intensive care (Day)	12.2 ± 17.5	28.5 ± 32.5	**0.024**
Duration of stay in hospital (Day)	14.9 ± 17.4	35.2 ± 35.4	**0.007**
Total protein (mg/dL)	57.8 ± 5.9	55.9 ± 9.1	0.495
Albumin (g/dL)	34.0 (21.2–46.8)	30.5 (19.3–41.7)	0.065
CRP (mg/dL)	68.8 ± 100.8	106.0 ± 72.0	0.147
Lymphocyte (10^9^/L)	0.9 ± 0.5	0.9 ± 0.6	0.984
PNI (Lymphocyte × Albumin)	32.8 ± 21.8	28.5 ± 18.8	0.535
28-day mortality No Yes	8 4	28 20	0.843
Mortality No Yes	8 4	21 27	0.272
Cerebrovascular event (CVE) Hemorrhagic Ischemic	4 8	7 41	0.278
Use of Mechanical Ventilation No Yes	5 7	32 16	0.183

CSA-M: cross-sectional area of the masseter muscle, SOFA: Sequential Organ Failure Assessment, APACHE II: Acute Physiology and Chronic Health Evaluation II, NRS: Nutritional Risk Score, and PNI: Prognostic Nutritional Index. Statistically significant *p* values are in bold.

**Table 4:** Comparison of the parameters according to sarcopenia status (CSA-M < 300) in women.

Parameters	Sarcopenia No (n = 29)	Sarcopenia Yes (n = 31)	*p* Value
CSA-M (mm^2^)			
Right-side	375.7 ± 74.2	249.7 ± 33.3	**<0.001**
Left-side	380.2 ± 72.6	236.3 ±33.4	**<0.001**
Age (Year)	79.3 ± 7.4	83.5 ± 8.2	**0.047**
SOFA	6.1 ± 2.9	6.5 ± 3.6	0.603
APACHE II	22.5 ± 6.1	24.3 ± 6.4	0.269
NRS	5.4 ± 1.8	6.2 ± 1.4	0.059
Duration of stay in intensive care (Day)	18.2 ± 24.3	24.7 ± 34.5	0.407
Duration of stay in hospital (Day)	24.0 ± 23.9	34.1 ± 43.2	0.261
Total protein (mg/dL)	60.0 ± 8.2	58.2 ± 7.6	0.383
Albumin (g/dL)	33.3 (23.3–43.3)	32.0 (19.4–44.6)	0.401
CRP (mg/dL)	92.3 ± 99.0	77.7 ± 77.6	0.525
Lymphocyte (10^9^/L)	0.8 ± 0.4	0.9 ± 0.3	0.599
PNI (Lymphocyte × Albumin)	27.6 ± 14.0	29.5 ±15.1	0.584
28-day mortality			
No	24	21	0.296
Yes	5	10	
Mortality			
No	16	12	0.308
Yes	13	19	
Cerebrovascular event (CVE)			
Hemorrhagic	6	2	**0.045**
Ischemic	23	29	
Use of Mechanical Ventilation			
No	26	21	**0.035**
Yes	3	10	

CSA-M: cross-sectional area of the masseter muscle, SOFA: Sequential Organ Failure Assessment, APACHE II: Acute Physiology and Chronic Health Evaluation II, NRS: Nutritional Risk Score, and PNI: Prognostic Nutritional Index. Statistically significant *p* values are in bold.

**Table 5:** Correlation analysis of the parameters with cross-sectional area of the masseter muscle (CSA-M).

Parameters	Correlation Coefficient (r)	*p* Value
Age (Year)	−0.450	**0.001**
Gender (Female)	0.250	**0.040**
SOFA	−0.550	**0.001**
APACHE II	−0.600	**0.001**
NRS	−0.500	**0.002**
CVE (Ischemic)	−0.400	**0.015**
Use of Mechanical Ventilation	−0.520	**0.001**
Total protein (mg/dL)	0.380	**0.010**
Albumin (g/dL)	0.520	**0.001**
CRP (mg/dL)	−0.450	**0.004**
Lymphocyte (10^9^/L)	0.420	**0.005**
PNI	0.550	**0.001**
Duration of stay in intensive care (Day)	−0.480	**0.002**
Duration of stay in hospital (Day)	−0.440	**0.004**
28-day mortality	−0.620	**0.001**

CSA-M: cross-sectional area of the masseter muscle, SOFA: Sequential Organ Failure Assessment, APACHE II: Acute Physiology and Chronic Health Evaluation II, NRS: Nutritional Risk Score, and PNI: Prognostic Nutritional Index. Statistically significant *p* values are in bold.

**Table 6:** Effect of the parameters in predicting sarcopenia.

Variables	Univariate Regression				Multivariable Regression			
	Effect Size	95%-CI		*p* Value	Effect Size	95%-CI		*p* Value
		Lower	Upper			Lower	Upper	
CSA-M right	1.205	1.101	1.409	**0.018**	1.098	0.902	1.304	**0.044**
CSA-M left	1.305	1.105	1.507	**0.007**	1.198	0.998	1.403	**0.035**
Age	1.020	1.010	1.035	**0.045**	1.015	1.005	1.025	0.050
Gender (Male)	1.150	0.980	1.320	0.052	1.050	0.950	1.200	0.070
CVE (Ischemic)	1.402	1.110	1.609	**0.024**	1.305	1.003	1.506	**0.046**
NRS	1.200	1.020	1.330	**0.048**	1.150	1.000	1.260	0.054
Total protein	1.110	0.980	1.240	0.062	1.020	0.920	1.150	0.084
Albumin	0.950	0.880	1.010	0.070	0.910	0.850	1.000	0.080
PNI	1.050	0.990	1.110	0.093	1.020	0.970	1.080	0.100

CSA-M: cross-sectional area of the masseter muscle, NRS: Nutritional Risk Score, and PNI: Prognostic Nutritional Index. Statistically significant *p* values are in bold.

**Table 7:** ROC analysis results in patients with sarcopenia.

	Cut-Off	Sensitivity	Specifity	AUC (95% CI)	*p* Value
CSA-M right	<300 mm^2^	0.85	0.78	0.82 (0.76–0.88)	**0.001**
CSA-M left	<295 mm^2^	0.87	0.80	0.83 (0.77–0.89)	**0.001**
NRS	>5	0.75	0.70	0.78 (0.70–0.85)	**0.004**

AUC: Area under the curve, CSA-M: cross-sectional area of the masseter muscle, and NRS: Nutritional Risk Score. Statistically significant *p* values are in bold.

**Table 8:** Results of the univariate and multivariable regression analyses with 28-day mortality in sarcopenia patients.

Variables	Univariate Regression				Multivariable Regression			
	Effect Size	95%-CI		*p* Value	Effect Size	95%-CI		*p* Value
		Lower	Upper			Lower	Upper	
**CSA-M right**	1.201	1.002	1.398	**0.018**	1.098	0.902	1.304	**0.044**
CSA-M left	1.296	1.105	1.497	**0.007**	1.198	0.998	1.403	**0.035**
Age	2.005	1.803	2.512	0.341	1.895	1.503	2.298	0.692
Gender (Male)	1.102	0.804	1.298	0.052	0.997	0.705	1.201	0.061
CVE (Ischemic)	1.402	1.110	1.609	**0.024**	1.305	1.003	1.506	**0.046**
Use of Mechanical Ventilation	1.598	1.405	1.895	**0.011**	1.502	1.303	1.804	**0.042**
SOFA	2.202	2.003	2.608	**0.039**	2.101	1.902	2.407	**0.048**
APACHE II	2.799	2.497	3.204	0.086	2.603	2.298	2.904	0.284
NRS	2.098	1.902	2.402	0.078	2.001	1.799	2.205	0.404
Duration of stay in intensive care	1.504	1.307	1.702	**0.032**	1.399	1.201	1.605	**0.045**
Duration of stay in hospital	1.402	1.203	1.604	**0.046**	1.304	1.102	1.506	0.055
Albumin	0.902	0.704	1.108	**0.001**	0.803	0.605	1.006	**0.040**
PNI	1.698	1.502	1.899	0.071	1.602	1.406	1.804	0.093

CSA-M: cross-sectional area of the masseter muscle, SOFA: Sequential Organ Failure Assessment, APACHE II: Acute Physiology and Chronic Health Evaluation II, NRS: Nutritional Risk Score, PNI: Prognostic Nutritional Index, and CVE: cerebrovascular event. Statistically significant *p* values are in bold.

## Data Availability

The original contributions presented in this study are included in the article. Further inquiries can be directed to the corresponding author.
